# The interplay between *Helicobacter pylori* and the gut microbiota: An emerging driver influencing the immune system homeostasis and gastric carcinogenesis

**DOI:** 10.3389/fcimb.2022.953718

**Published:** 2022-08-15

**Authors:** Farzaneh Fakharian, Behnoush Asgari, Ali Nabavi-Rad, Amir Sadeghi, Neda Soleimani, Abbas Yadegar, Mohammad Reza Zali

**Affiliations:** ^1^ Foodborne and Waterborne Diseases Research Center, Research Institute for Gastroenterology and Liver Diseases, Shahid Beheshti University of Medical Sciences, Tehran, Iran; ^2^ Basic and Molecular Epidemiology of Gastrointestinal Disorders Research Center, Research Institute for Gastroenterology and Liver Diseases, Shahid Beheshti University of Medical Sciences, Tehran, Iran; ^3^ Gastroenterology and Liver Diseases Research Center, Research Institute for Gastroenterology and Liver Diseases, Shahid Beheshti University of Medical Sciences, Tehran, Iran; ^4^ Department of Microbiology, Faculty of Biological Sciences and Technology, Shahid Beheshti University, Tehran, Iran

**Keywords:** *Helicobacter pylori*, gut microbiota, gastric carcinogenesis, immune system homeostasis, probiotics, immunotherapy

## Abstract

The human gut microbiota are critical for preserving the health status because they are required for digestion and nutrient acquisition, the development of the immune system, and energy metabolism. The gut microbial composition is greatly influenced by the colonization of the recalcitrant pathogen *Helicobacter pylori* (*H. pylori*) and the conventional antibiotic regimens that follow. *H. pylori* is considered to be the main microorganism in gastric carcinogenesis, and it appears to be required for the early stages of the process. However, a non-*H. pylori* microbiota profile is also suggested, primarily in the later stages of tumorigenesis. On the other hand, specific groups of gut microbes may produce beneficial byproducts such as short-chain fatty acids (acetate, butyrate, and propionate) that can modulate inflammation and tumorigenesis pathways. In this review, we aim to present how *H. pylori* influences the population of the gut microbiota to modify the host immunity and trigger the development of gastric carcinogenesis. We will also highlight the effect of the gut microbiota on immunotherapeutic approaches such as immune checkpoint blockade in cancer treatment to present a perspective for further development of innovative therapeutic paradigms to prevent the progression of *H. pylori*-induced stomach cancer.

## 1 Introduction

*Helicobacter pylori* (*H. pylori*) is a Gram-negative spiral-shaped pathogen colonizing the host stomach and correlated with various gastric disorders such as chronic active gastritis, peptic ulcer disease (PUD), mucosa-associated lymphoid tissue (MALT) lymphoma and gastric carcinoma (Yamade et al., 2011; Sheh and Fox, 2013; Hooi et al., 2017). Gastric cancer is the third most prevalent cause of cancer-related death in both genders globally (Rawla and Barsouk, 2019; Choi et al., 2020). It has been suggested that more than 50% of the world’s population is infected with *H. pylori*, but it remains asymptomatic in the majority of the general population (Sheh and Fox, 2013; Hooi et al., 2017). The severity of *H. pylori* infection largely relies on the host genetics, immune response, and microbiome composition, as well as on bacterial virulence, and environmental conditions (Ding et al., 2010; Rawla and Barsouk, 2019). It has been long assumed that the human stomach is sterile owing to its harsh acidic environment; however, the discovery of *H. pylori* in 1982 revealed that the human gastric tissue holds a core microbiome (Warren and Marshall, 1983; Kusters et al., 2006).

It is now well documented that a normal gut microbiome is critically associated with the modulation of the host immune response, energy metabolism, and pathogen colonization resistance (Belkaid and Hand, 2014; Pascale et al., 2019). *H. pylori* can modify the α-diversity of the normal gastric microbiome that can result in dysbiosis and the development of gastric carcinoma (Hooi et al., 2017; Yang et al., 2019). The imbalance in the gastric microbiome community can result in continuous stimulation of the host immune system, which can in turn cause chronic inflammation of gastric mucosa or trigger tumor growth (Nasr et al., 2020; Zhang and Pan, 2020). Development of immunotherapy agents such as immune checkpoint inhibitors (ICIs) and also probiotic co-supplementation along with antibiotic regimens have been suggested as promising modalities for the treatment of *H. pylori* infection and gastric carcinoma (Qureshi et al., 2019; Rezasoltani et al., 2021). In recent years, cancer immunotherapy achieved brilliant breakthroughs in the field of clinical oncology by extending the lives of patients suffering from rapidly lethal tumors. The significance of immunotherapy in cancer research and treatment will develop expeditiously in the next decades as novel treatment strategies, and brand new druggable targets are expected to be discovered accordingly (Waldman et al., 2020). Here, we intend to highlight the latest and available literature regarding the role of *H. pylori* infection in perturbation of the gut microbiota and the probable role of dysbiosis in gastric carcinogenesis and tumorigenesis. We further discuss the reports for crosstalk between the gut microbiome and immunotherapeutic approaches in gastric carcinoma.

### 1.1 The human gut microbiota

It has been established that the human microbiota is the collection of a variety of microorganisms including bacteria, archaea, fungi, viruses, and parasites, which occupy various niches on or within human tissues and biofluids. The impact of these microbes on the human body may be beneficial or harmful (Stasiewicz and Karpinski, 2021). It is established for many years that the human body is hosting remarkable microbiota and includes 10-fold more microorganisms (10^14^) than human cells (Savage, 1977). These microorganisms are found inside and outside the human body exposed to the external environment, including the oral cavity, skin, respiratory, urogenital, and gut tracts. Among these potential niches, the gut has the highest density and variety of microbiota due to its ideal growth conditions (Ohland and Jobin, 2015). The complex ecosystem residing inside or passing through the digestive tract is termed the gut microbiome (Kastl et al., 2020). The oral cavity is enriched with over 700 bacterial species and thereby has the second largest level of α-diversity after that of the gut microbiome *(* Deo and Deshmukh, 2019 *)*. A recent study has demonstrated that in a healthy oral cavity, the predominant bacterial genera are 12 Gram-positive microorganisms including *Corynebacterium*, *Abiotrophia*, *Peptostreptococcus*, *Streptococcus*, *Pseudoramibacter*, *Stomatococcus*, *Actinomyces*, *Propionibacterium*, *Bifidobacterium*, *Eubacterium*, *Lactobacillus*, and *Rothia*, as well as Gram-negative bacteria including *Eikenella*, *Moraxella*, *Neisseria*, *Prevotella*, *Veillonella*, *Campylobacter*, *Capnocytophaga*, *Treponema*, *Desulfobacter*, *Desulfovibrio*, *Fusobacterium*, *Hemophilus*, *Leptotrichia*, *Selemonas*, *Simonsiella*, and *Wolinella* (Deo and Deshmukh, 2019). It has been well understood that the oral microbiome are associated with multiple oral diseases. This association may arise from the ability of many oral bacteria to influence the oral inflammatory microenvironment (Lu et al., 2019).

Currently, the esophagus is suggested to contain a diverse microbiota, yet our understanding of the esophageal microbial composition and activity remains limited compared to that of the intestinal microbiota (Deshpande et al., 2018). However, several pieces of research have exhibited the correlation between the esophageal microorganisms and multiple esophageal illnesses following the advances in next-generation sequencing (NGS) techniques (Park and Lee, 2020). Moreover, many researchers have explored the structure of the esophageal microbiome in a normal esophagus and various esophageal disorders. *Streptococcus* is the dominant bacterial genus in the esophagus of a normal host followed by *Haemophilus*, *Neisseria*, *Prevotella*, and *Veillonella (* Jung et al., 2022 *)*. Nevertheless, Gram-negative bacteria such as *Prevotella*, are more prevalent in an abnormal esophagus, such as in esophageal cancer, Barrett’s esophagus, gastroesophageal reflux disease (GERD), and eosinophilic esophagitis (EoE) (Park and Lee, 2020).

As mentioned above, the gastric microbiota is composed of several microorganisms, which have been presented in the human body since birth (Schmidt et al., 2018; Altveş et al., 2019). Owing to the acidic condition of the stomach, the gastric environment was previously considered as an unsuitable niche for the colonization of bacteria (Ansari and Yamaoka, 2017). The discovery of *H. pylori* by Marshall in 1982 revealed that various microbes could reside in the stomach (Warren and Marshall, 1983; Garcia-Castillo et al., 2018). The most abundant gastric microbiota phyla are Proteobacteria and Firmicutes, followed by Bacteroides, Actinobacteria, and Fusobacteria (Cao and Yu, 2015; Rinninella et al., 2019).

The gut microbial population can communicate with one another and their hosts by switching genes on and off via metabolically active chemicals (Gao et al., 2018b). The gut microbiota are involved in human metabolism by producing enzymes, which are not expressed by the human genome, such as those involved in the breakdown of polysaccharides and polyphenols (Rowland et al., 2018). Dietary fibers and other undigested materials are consumed by gut colonizing bacteria, which can create short-chain fatty acids (SCFA). In a mutualistic connection, the microbes benefit the host cell physiology, whereas the host supplies an appropriate habitat for the bacteria to reside and grow (Rinninella et al., 2019; Pascale et al., 2019). The presence of the gut microbiota is vital for preserving human health, absorption of nutrients, synthesis of some vitamins, drug metabolism, immunomodulation, and defense against pathogens colonization (Collado et al., 2009; Cao and Yu, 2015; Magne et al., 2020).

The gut microbiota are very dynamic and could be affected by different factors such as host lifestyle, antibiotic therapy, long-term proton pump inhibitors (PPIs) consumption, and *H. pylori* infection (Schmidt et al., 2018; Zhang et al., 2019). Perturbation in the gut homeostasis has been correlated with a wide spectrum of behavior to mental disorders (Moran-Ramos et al., 2020). *H. pylori* infection can increase the stomach pH and induce chronic inflammation of the gut epithelium; therefore, affect the microenvironment of the stomach, causing extensive changes in the diversity and structure of the gut microbiota (Mohammadi et al., 2020).

## 2 *H. pylori* and the gastric microbiota

### 2.1 The gastric microbiota in a healthy stomach

Prior to the discovery *of H. pylori*, the stomach was assumed to be free of microbes owing to its harsh and acidic environment (Yang et al., 2013). As the culture-based techniques are the keystone of microbiological research, *H. pylori* was presumed as the sole microorganism with the capacity of replication in the severe gastric environment for many decades (Stockbruegger, 1985). Based on culture-independent approaches, such as NGS, fluorescent *in situ* hybridization (FISH), and dot-blot hybridization analysis of the gastric microbiome, it is now revealed that the bacteria in the human stomach are more dense and diverse than previously assumed (Li et al., 2009).

In more than 20% of the NGS-based studies, Tenericutes, Bacteroidetes, Firmicutes, Actinobacteria, Spirochetes, Proteobacteria, Fusobacteria, and TM7 are among the bacterial phyla constituting the normal gastric microbiota. Moreover, *Neisseria*, *Hemophilus*, *Fusobacterium*, *Prevotella*, *Veillonella*, and *Streptococcus* were the six most commonly reported genera of the gastric microbiota in a healthy stomach (Rajilic‐Stojanovic et al., 2020). Another study performed in Sweden demonstrated that Firmicutes (42%), Bacteroidetes (24%), Proteobacteria (17%), Actinobacteria (7%), and Fusobacteria (6%) were allocated to the five most common phyla in the gastric mucosa of *H. pylori*-negative individuals. Additionally, *Streptococcus* (Firmicutes, 24%), *Prevotella* (Bacteroidetes, 23%), *Veillonella* (Firmicutes, 6%), *Fusobacterium* (Fusobacteria, 5%), *Haemophilus* (Proteobacteria, 4%), *Neisseria* (Proteobacteria, 4%), and *Gemella* (Firmicutes, 4%) were reported as the dominant genera among the inherent gastric microbiota (Ndegwa et al., 2020). These findings are consistent with earlier research that showed Proteobacteria, Actinobacteria, Firmicutes, and Bacteroidetes as the most common taxa in the gastric mucus layer, yet their proportions vary between different studies and geographical regions. Although *Prevotella*, *Veillonella*, *Streptococcus*, *Neisseria*, *Fusobacterium*, and *Haemophilus* are the main genera among the gastric microbiota, data on the variety of the human microbiome are not uniform, indicating regional variances in the microbial abundance (Ozbey et al., 2020; Rajilic‐Stojanovic et al., 2020; Miftahussurur et al., 2020).

### 2.2 Impact of *H. pylori* on the gastric microbiota

It has been demonstrated that *H. pylori* possesses well-developed adaptation mechanisms that accelerate bacterial growth in the severe acidic niche of the stomach and promote the incidence of developing long-term infection (Ansari and Yamaoka, 2017). *H. pylori* is the most frequent microorganism detected in the gastric microbial ecology of an infected individual, accounting for 40% to 90% of the gastric microbiota (Chen et al., 2021). In general, *H. pylori* colonization results in a substantial downregulation in the overall α-diversity of the gastric microbiome. According to a recent study on the gastric microbial structure, the normal stomach demonstrated a significantly higher microbial α-diversity using the Shannon index analysis, compared to individuals with chronic *H. pylori* gastritis and atrophic gastritis (Ndegwa et al., 2020). It has been further indicated that *H. pylori* oncoproteins, especially cytotoxin-associated gene A (CagA), can disturb the gastric environment and trigger microbial dysbiosis (Ansari and Yamaoka, 2017).

Recently, it has been reported that the gastric microbiota of *H. pylori*-positive and *H. pylori*-negative subjects are enriched by the common phyla, albeit with different rates of relative abundance (Vasapolli et al., 2019). Proteobacteria (68.7%) are the most prevalent phyla in *H. pylori*-positive individuals, followed by Firmicutes (14.7%), Bacteroidetes (8.3%), and Actinobacteria (6%). However, in an *H. pylori*-negative population, the proportions of the gastric microbiota were reported as Proteobacteria (52.6%), Firmicutes (26.4%), Bacteroidetes (12%), and Actinobacteria (6.4%) (Llorca et al., 2017; Li et al., 2017). Furthermore, *H. pylori* infection will disturb the Firmicutes/Bacteroidetes (F/B) ratio and probably change the physiological state of the individual, especially in asymptomatic subjects (Sheh and Fox, 2013; Khosravi et al., 2016; Kienesberger et al., 2016). Firmicutes, Bacteroidetes, Actinobacteria, Cyanobacteria, and Fusobacteria were among the dominant commensal microbiota enriched after *H. pylori* eradication. Specific genera that increased among the gastric microbiota following *H. pylori* eradication included *Prevotella*, *Gemella*, *Porphyromonas*, *Alloprevotella*, *Veillonella*, *Neisseria*, *Streptococcus*, *Rothia*, and *Haemophilus* (Guo et al., 2020).

Due to the connection of the stomach to the esophagus, oral cavity, and duodenum, bacteria from the mouth, pharynx, nose, respiratory tract, esophagus, and small intestine can enter the stomach. Therefore, the occurrence of several gastrointestinal diseases, such as esophagitis, Barrett’s esophagus, esophageal carcinoma, gastritis, gastric cancer, small intestine bacterial overgrowth, irritable bowel syndrome (IBS), and celiac disease depends on the structure of the gut microbiota (Wang and Yang, 2013). Aside from the influence of *H. pylori* on the gastric microbial structure, several investigations have exhibited the essential impact of this bacterium on the gut microbial community. It has also been demonstrated that there are multiple host-microbe and microbe-microbe interactions between *H. pylori* and other bacterial species found in the digestive tract (Engstrand and Lindberg, 2013). It has been further revealed that *H. pylori* modifies the gut microbial community in three ways: first by modifying the level of stomach acidity (Goddard and Spiller, 1996; Navabi et al., 2013; Espinoza et al., 2018), second by supplying substrates favorable for the colonization of other bacterial species (Bauerfeind et al., 1997; Ziebarth et al., 1997), and third by changing the lifestyle and diet patterns of the host (Dong et al., 2017; Noto and Peek Jr, 2017). The correlation between the gut microbiota and *H. pylori* infection or gastric carcinoma in humans is yet to be fully elucidated (Gao et al., 2018a; Park et al., 2019).

### 2.3 *H. pylori*, the gut microbiota, and gastric cancer

Approximately 75% of all gastric cancer subjects are caused by *H. pylori* infection, which significantly contributes to the progression of low-grade MALT lymphoma, a subcategory of primary gastric lymphoma, representing about 30-40% of extranodal lymphomas (Fehlings et al., 2012; Noto and Peek Jr, 2017). *H. pylori* colonizes the gastric mucosa using different virulence factors such as *cag* pathogenicity island (*cag*PAI), CagA, vacuolating cytotoxin A (VacA), outer membrane proteins (OMPs), peptidoglycan, IceA as well as several adherence factors such as BabA, SabA, and OipA, which are involved in the carcinogenesis of gastric tissue (Cao and Yu, 2015; Espinoza et al., 2018; Ohno and Satoh-Takayama, 2020). During *H. pylori* infection, VacA stimulates CagA accumulation in the gastric epithelial cells. *cag*PAI encodes a syringe-like structure named type IV secretion system (T4SS) to facilitate the efficient delivery of CagA oncoprotein into the host cell. It is well documented that *H. pylori* strains expressing CagA are substantially correlated with high grades of inflammation in the gastric epithelium, resulting in chronic gastritis and gastric atrophy (Cao and Yu, 2015). While *H. pylori* bacteria are dominated in the stomach, their lipopolysaccharide (LPS) and surface proteins are released in the gastric lamina propria, after which they might provoke macrophages to increase the expression of pro-inflammatory cytokines such as interleukin-1β (IL-1β), IL-8, IL-17, tumor necrosis factors-α (TNF-α), and ultimately increases the risk of developing *H. pylori*-induced gastric carcinoma (Naito and Yoshikawa, 2002; Fehlings et al., 2012; Guo et al., 2013).

IL-8, a potent chemoattractant cytokine secreted by a plethora of tissue and blood cells, may promote the production of CD11b/CD18 in leukocytes and intercellular adhesion molecule-1 (ICAM-1) in endothelial cells, leading to the transmigration of leukocytes from the bloodstream to the gastric mucosa (Espinoza et al., 2018). Alteration in the gut microbial structure can elevate the secretion levels of systemic and local pro-inflammatory mediators as well as the expression of cancer-related genes, which ultimately contribute to the progression of gastric diseases (Zhang et al., 2019).

#### 2.3.1 The oral microbiota

The dynamic and polymicrobial oral microbiota directly affect the disease status of dental caries and periodontal diseases. There is solid evidence that the gastric microbiota of oral origin may play a role in gastric cancer development and progression. In spite of this, there is still much controversy regarding the relationship between the oral microbiota and gastric cancer progression as well as the role of *H. pylori* pathogenesis in this interaction. According to recent studies, patients with stomach cancer had more complicated oral bacteria than the normal control population. This might be due to the weak immunity of the patients with gastric cancer compared to healthy individuals (Sun et al., 2018; Bakhti and Latifi-Navid, 2021).

The presence of *H. pylori* in the gastric mucosa significantly influences gut colonization dynamics. It has been shown that *H. pylori* interacts with the oral microbiota and may even colonize in the oral cavity. Moreover, a significant increase in the α-diversity of the oral microbiota has been detected in *H. pylori-*positive individuals compared to *H. pylori*-negative individuals (Krzyzek and Gosciniak, 2018).

A recent study showed that the development of esophageal and gastric cancer may be associated with the oral pathobiont *Fusobacterium* spp., which isolated from dental plaques (*F. nucleatum* and *F. periodontium*) (Alon-Maimon et al., 2022). Dental plaque produces a key signaling substance called autoinducer-2 (AI-2), which is a chemorepellent agent, stimulating dispersion of *H. pylori* aggregates and biofilms and starting negative chemotaxis against the signal source (Anderson et al., 2015). Between the *H. pylori*-positive and *H. pylori*-negative groups, there were significant differences in the genera *Actinomyces*, *Neisseria*, *Granulicatella*, *Helicobacter*, *Veillonella*, *Streptococcus*, *Fusobacterium*, and *Prevotella* in the oral cavity. Additionally, in the bacterial profile of saliva or plaque of the patients infected with *H. pylori*, the higher relative abundance of *Veillonella, Prevotella*, *Lactobacillus*, *Aggregatibacter*, *Streptococcus*, and *Megasphaera*, and a lower proportion of *Leptotrichia*, *Rothia*, *Capnocytophaga*, *Campylobacter*, *Tannerella*, and *Granulicatella* was associated with stomach cancer (Dicksved et al., 2009). Furthermore, during the process from gastritis to stomach cancer, *Lactobacillus* bacteria may thus grow over other microorganisms in the oral cavity (Li et al., 2021). A study by Wu et al. showed that microbiota coating the tongue differ between healthy people and gastric cancer patients. Results from this study revealed that a higher relative abundance of Firmicutes and a lower relative abundance of Bacteroidetes were associated with the risk of developing gastric carcinoma (Wu et al., 2018). The prevalence of periodontal pathogens in the oral cavity specifically *Treponema denticola*, *Tannerella forsythia*, and *Actinobacillus actinomycetemcomitans* are currently considered significant risk factors for the development of precancerous stomach lesions (Sun et al., 2017). There are numerous fundamental mechanisms through which the oral bacteria can cause upper gastrointestinal cancer, such as releasing carcinogenic chemicals, stimulating chronic inflammation, and increasing the levels of certain bacterial metabolic pathways (Bakhti and Latifi-Navid, 2021).

#### 2.3.2 The esophageal microbiota

The microbial composition of a normal esophagus is relatively conserved without distinct differences between the upper, middle, and lower esophagus. As with the oral microbiota, the esophageal microbiota comprises six major phyla, which include Firmicutes, Bacteroides, Actinobacteria, Proteobacteria, Fusobacteria, and TM7 (Gillespie et al., 2021). Alterations in the esophageal microbiome may be linked to the esophageal mucus injury and consequently disrupts the integrity of the epithelial barrier and results in the translocation of other microbes. There is a hypothesis that stands for the protective role of *H. pylori* against Barrett’s esophagus and esophageal adenocarcinoma (EAC), which might be contributed to the gastric acid output reduction (Anderson et al., 2008; Polyzos et al., 2018).

The dysbiotic status of the esophageal microbiota may play a crucial role in disturbing the epithelial barrier, causing chronic inflammation, inducing DNA damage, and promoting gastrointestinal carcinogenesis. Despite the lack of clarity around the direct contribution of the esophageal microbiota to gastric cancer, mounting data suggest that considerable changes in the esophageal microbial community may contribute to inflammation-induced gastric carcinogenesis (Sharma et al., 2022).

#### 2.3.3 The gastric microbiota

It has been shown that Actinobacteria, Bacteroidetes, and Firmicutes are the main phyla in the stomach microbiota of *H. pylori*–negative subjects (Zhang et al., 2019). In contrast, the colonization of *H. pylori* in the host stomach will lead to the enrichment of Spirochetes and Proteobacteria phyla. Many carcinogenic metabolites such as N-nitroso compounds produced by *Staphylococcus*, *Lactobacillus*, and *Escherichia coli* are involved in various disadvantageous cellular mechanisms, which can trigger inflammation and tumor angiogenesis (Mowat et al., 2000; Wang et al., 2016; Pero et al., 2019). Furthermore, the balance of stomach pH may perturb during abnormal or disease states and leads to hypochlorhydria, which is correlated with higher levels of intragastric nitrite production and a greater risk of gastric cancer development. Consequently, a decreased gastric acidity elevates the risk of bacterial overgrowth and alters the indigenous community of the gastric microbiota. This will promote the colonization of pathobionts and those microbes with nitrosating capability, which are not frequently cultured from the microbial community of a healthy individual (Hunt et al., 2015). The promotion in the transformation of nitrogen compounds into carcinogenic N-nitroso compounds is promoted by chronic H2 receptor antagonists (H2RA) therapy that accelerate the colonization of nitrosating bacteria (Muscroft and Keighley, 1981; Stockbrugger et al., 1982; Fisher and Fisher, 2017; Piscione et al., 2021). As ascorbic acid is a powerful inhibitor of nitrosation reaction, these chemical reactions may take place when the stomach has the lowest gastric levels of ascorbic acid (Toh and Wilson, 2020). Furthermore, the rate of these chemical nitrosation reactions increases when the pH of the stomach is less than 4. In addition to *H. pylori*, some non-*H. pylori* bacteria were found to increase atrophy in patients following acid-suppressive medications (Li and Perez Perez, 2018). Based on a recent report, researchers discovered that patients taking acid-suppressing drugs such as H2 receptor blockers (H2RBs) or PPIs have a three-fold higher risk of developing infectious diarrhea compared to non-recipients (Martinsen et al., 2019).

Decreases in the ratio of beneficial *Clostridium* clusters XIVa and IV, including several butyrate-producing bacteria, have been also reported in multiple elderly individuals. As previously documented, the gastric cancer microbiota largely consists of several strains of the genera *Streptococcus*, *Lactobacillus*, *Veillonella*, and *Prevotella* (Aviles-Jimenez et al., 2014). Among the *Streptococcus* species, *Streptococcus mitis* and *Streptococcus parasanguinis* were found to be the predominant bacteria in the gastric mucosa of patients with gastric carcinoma (Dicksved et al., 2009). In another study, an overgrowth in Proteobacteria (including the genera *Phyllobacterium*, *Achromobacter*, and the families Xanthomonadaceae and Enterobacteriaceae) and the genera *Lactobacillus*, *Clostridium*, and *Rhodococcus*, was observed in gastric carcinoma (Ferreira et al., 2018). Moreover, Coker et al. indicated that the enrichment of *Slackia exigua*, *Parvimonas micra*, *Fusobacterium nucleatum*, *Dialister pneumosintes*, *Prevotella intermedia*, *Prevotella oris*, and *Catonella morbi* bacteria in the gastric microbiota elevates the risk of developing gastric cancer (Coker et al., 2018). Wang et al. in 2016 demonstrated that the microorganisms most frequently isolated from gastric cancer tissues were *Lactobacillus*, *Shigella, Escherichia*, *Nitrospirae*, *fungerum*, *Burkholderia* and *Lachospiraceae* (Stockbruegger, 1985). Rodríguez et al. concluded that *Veilonella*, *Lactococcus*, and Fusobacteriaceae (*Fusobacterium* and *Leptotrichia*) were among the bacterial taxa dominated in gastric cancer (Castaño-Rodríguez et al., 2017). Cavadas et al. demonstrated that a decrease in the proportion of *Rhodococcus*, *Phyllobacterium*, and *Staphylococcus* genera was correlated with a reduction in the microbial diversity of cancer cases, which was counterbalanced by enrichment in *Bacillus*, *Enterobacter*, *Fusobacterium*, and *Sutterella* genera (Cavadas et al., 2020). *Lactobacillus*, *Paeniglutamicibacter*, *Glutamicibacter*, *Helicobacter*, *Enterococcus*, and *Carnobacterium* genera were linked to gastric carcinoma in a case-control study in Mongolian patients (Gantuya et al., 2020). It has been further suggested that as precancerous lesions progressed to dysplasia and gastric cancer, the relative abundance of *Capnocytophaga*, *Bacillus*, and *Prevotella* bacteria increased, yet the relative abundance of *Helicobacter* genus decreased (Kadeerhan et al., 2021). Recently, Deng et al. (Deng et al., 2021) found an important but not statistically significant increase in Proteobacteria (mostly Pseudomonadales) in biopsies from antral gastric cancer compared to biopsies from antrum gastritis (from 38% to 60%) that was followed by a substantial reduction in Actinobacteria (from 15% to less than 1% of total sequencing reads). Also, they detected that Pseudomonadales and Erysipelotrichales have been enriched in individuals with *H. pylori*-negative antral gastric cancer, whereas Neisseriales were enriched in individuals with *H. pylori*-positive antral gastric carcinoma. Li et al. exhibited that the presence of *Haemophilus*, *Serratia*, *Neisseria*, and *Stenotrophomonas* was correlated with the development of gastric cancer (Li et al., 2017). Based on the recent findings that all types of gastric tumors are associated with an elevated enrichment of Enterobacteriaceae and a decreased proportion of Lactobacillaceae and *Oscillibacter*, it has been suggested that the correlation of higher Enterobacteriaceae abundance with gastric malignancies might be potentially introduced as a biomarker of gastric cancer (Sarhadi et al., 2021). These findings contradicted the results from Danish and Lithuanian studies, in which they found that the proportion of bacteria from the genera *Gemella*, *Lactobacillus*, *Streptococcus*, and *Enterococcus* (all Firmicutes) was higher in patients suffering from gastric cancer than in dyspeptic subjects, whereas the abundance of bacteria from the genera *Staphylococcus*, *Actinomyces*, and *Corynebacterium* was lower (Spiegelhauer et al., 2020). Another study revealed that *H. pylori*-infected individuals had significantly higher colonization of Proteobacteria bacteria and lower colonization of Actinobacteria, *Streptococcus*, *Lactobacillus*, *Veillonella*, and *Prevotella* bacteria, which may promote inflammation that could potentially accelerate cancer development (Aviles-Jimenez et al., 2014). In agreement with the aforementioned data, another investigation evaluated by Eun et al. reporting that *Streptococcus*, *Lactobacillus*, *Veillonella*, and *Prevotella* were the main bacterial genera in the gastric mucosa of individuals with stomach cancer (Eun et al., 2014). Wang et al. exhibited that *Nitrospirae*, *Escherichia*, *Lactobacillus*, *Shigella*, *Burkholderia*, and uncultured Lachnospiraceae were the main gastric microbiome found in individuals with gastric adenocarcinoma (Wang et al., 2016). A recent study speculated that the enrichment of Actinobacteria, *Staphylococcus epidermidis* and nitrosating/nitrate-reducing bacteria may lead to gastric cancer development. Furthermore, an enhancement in the population of *Lactobacillus*, *Clostridium colicanis*, and *F. nucleatum* bacteria represents diagnostic biomarkers for gastric cancer progression (Hsieh et al., 2018). *Aggregatibacter*, *Neisseria*, *Streptococcus oralis*, *Alloprevotella*, *Streptococcus mitis, Streptococcus pneumoniae*, and strain *Porphyromonas endodontalis.t_GCF_000174815* are further suggested as the major stomach microbiome engaged in the development of gastric carcinoma (Hu et al., 2018). Additionally, several other studies have explored the effect of *H. pylori* infection on the diversity and structure of the gastric microbiome. In agreement with the aforementioned studies, the α-diversity of the gastric microbiota was reported higher in subjects with a healthy stomach mucosa, followed by the individuals with gastritis, intestinal metaplasia, precancerous lesions, and gastric cancer. This might suggest that disease progression reduces the mucosal microbial diversity and richness (Miftahussurur et al., 2020; Ndegwa et al., 2020; Rajilic‐Stojanovic et al., 2020).

**Figure 1:**
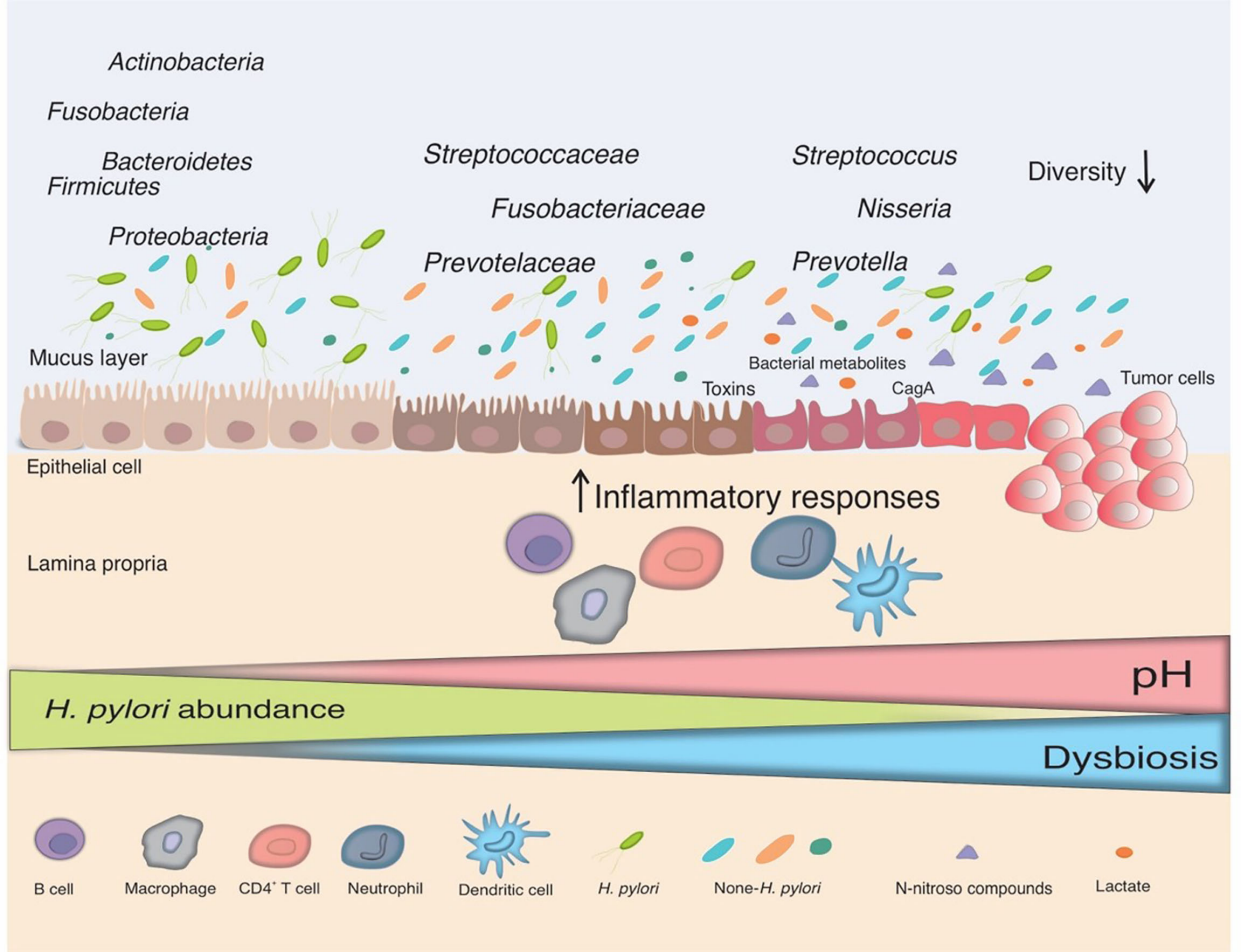
The interplay between *H. pylori* and the gut microbiota. Infection with *H. pylori* causes and maintains an inflammatory response in the gastric mucosa, which leads to the loss of acid-secreting parietal cells and an elevation in gastric pH in certain infected people. *H. pylori* colonization declines in this changing environment and bacteria from other parts of the gut colonize the gastric niche, resulting in gut dysbiosis. Non-*H. pylori* bacteria promote gastric carcinogenesis through their own characteristics and microbial metabolites, such as N-nitroso compounds and lactate. The main putative mechanisms include induction of inflammatory response, modulation of the immune response, induction of DNA damage, and development of carcinogenesis.

As depicted in **Figure 1**, the overall evenness and diversity of the gastric microbiota in the lumen of patients with gastric carcinogenesis will decrease in comparison with healthy individuals. Some substantial alterations in the gastric microbiota composition of individuals suffering from gastric cancer are presented in **Table 1**.

**Table 1:** Studies published on the gastric microbiome analysis in patients with gastric cancer.

Authors/Year	Participants	Sample type	Microbiome analysis method	Taxon differences	Reference
Dicksved et al./2009	10 GC	GB	16S rRNA gene sequencing (T-RFLP)	↑ *Streptococcus*, *Lactobacillus*, *Veillonella, Prevotella* ↓ *H. pylori*	(Dicksved et al., 2009)
Sjöstedt et al./1985	NA	GJ	16S rRNA gene sequencing	↑ Proteobacteria, Firmicutes (genera *Veilonella* and *Streptococcus*, *Bifidobacterium*, *Lactobacillus* group	(Sjostedt et al., 1985)
Aviles-Jimenez et al./2014	5 intestinal-type GC	GB	Microarray G3 PhyloChip	↑ *Lactobacillus*, *Lachospiraceae* ↓ *Porphyromonas*, *Neisseria*, *S. sinensis*	(Aviles-Jimenez et al., 2014)
Eun et al./2014	11 GC	GM	16S rRNA gene sequencing	↑ *Streptococcus*, *Lactobacillus*, *Veillonella, Prevotella*	(Eun et al., 2014)
Maldonado-Contreras et al./2011	NA	GB	16S rRNA gene sequencing	↑ Proteobacteria, Spirochetes, Acidobacteria ↓ Actinobacteria, Bacteroidetes, Firmicutes	(Maldonado-Contreras et al., 2011)
Wang et al./2016	6 GC, 6 CG	GM	16S rRNA gene sequencing	↑ *Lactobacillus*, *Escherichia*, *Shigella*, *Nitrospirae*, *Burkholderia*, *Fungerum*, *Lachospiraceae*	(Wang et al., 2016)
Jo et al./2016	34 GC, 29 control	GM	16S rRNA gene sequencing	↑ *Actinobacteria*, *S. epidermidis* ↑ Nitrosating/nitrate reducing bacteria	(Jo et al., 2016)
Castaño-Rodriguez et al./2017	12 GC, 20 dyspepsia	GB	Illumina MiSeq platform targeting the 16S rDNA	↑ *Lactococcus*, *Veilonella*, *Fusobacterium*, *Leptotrichia*	(Castaño-Rodríguez et al., 2017)
Li et al./2017	9 GC	GB	Illumina MiSeq platform targeting the 16S rRNA	↑ *Flavobacterium*, *Klebsiella*, *Serratia marcescens*, *Stenotrophomonas*, *Achromobacter*, *Pseudomonas*, *Delftia*, *Ralstonia*, *Rhizobium*, *Elizabethkingia meningoseptica*, *Methyloversatiis*, *Cytophagacea*	(Li et al., 2017)
Parsons et al./2017	95 GC	GB	16S rRNA gene sequencing	↑ *Helicobacteraceae*, *Streptococcaceae*, *Fusobacteriaceae*, *Prevotellaceae* ↓ *Tannerella (Escherichia*, *Shigella*, *Salmonella*, *Treponema*, *Prevotella)*	(Parsons et al., 2017)
Yu et al./2017	160 GC	GB	16S rRNA gene sequencing	↑ Bacteriodetes, Firmicutes, Fusobacteria, Spirochetes ↓ Proteobacteria	(Yu et al., 2017)
Ferreira et al./2018	54 GC, 81 CG	GM	16S rRNA gene sequencing	↑ *Achromobacter*, *Lactobacillus*, *Clostridium*, *Rhodococcus*, *Citrobacter*	(Ferreira et al., 2018)
Coker et al./2018	20 GC, 21 superficial gastritis, 23 atrophic gastritis, 17 IM	GM	16S rRNA gene sequencing	↑ *Peptostreptococcus stomatis*, *S. anginosus*, *Parvimonas micra*, *S. exigua*, *D. pneumosintes* ↓ *Vogesella*, *Comamonadaceae*, *Acinetobacter*	(Coker et al., 2018)
Hu et al./2018	6 GC, 5 SG	GB	16S rRNA gene sequencing	↑ *Neisseria*, *Alloprevotella*, *Aggregatibacter*, *S. mitis*, *Porphyromonoas endodontalis* ↓ *Sphingobium yanoikuyae*	(Hu et al., 2018)
Hsieh et al./2018	9 gastritis, 7 IM, 11 GC	GB	16S rRNA gene sequencing	↑ *Lactobacillus*, *C. colicanis*, *F. nucleatum*	(Hsieh et al., 2018)
Park et al./2019	55 GC, 19 IM, 62 CG	GB	16S rRNA gene sequencing	↑ *Rhizobiales* ↑ *Cyanobacteria* in *H. pylori*–negative	(Park et al., 2019)
Gunathilake et al./2019	288 GC, 288 control	GB	16S rRNA gene sequencing	↑ *P. copri*, *P. acnes*	(Gunathilake et al., 2019)
Spiegelhauer et al./2020	22 dyspepsia, 12 GC	GB	16S rRNA gene sequencing	↑ *Lactobacillus*, *Prevotella* spp., *Enterococcus* spp., *Streptococcus* ↓ *Actinomyces*	(Spiegelhauer et al., 2020)
Ndegwa et al./2020	316 GC	GB	16S rRNA gene sequencing	↑ *Acinetobacter*, *Aeromonas*, *Asticcacaulis*, *Atopobium*, *DelftiaEwingella*, *Kocuria*, *Brevibacterium*, *Delftia*, *Halomonas*, *Kocuria*, *Lactococcus*, *Variovorax*	(Ndegwa et al., 2020)
Cavadas et al./2020	164 healthy, 137 GC	GB	16S rRNA gene sequencing	↑ *Bacillus*, *Enterobacter*, *Fusobacterium*, *Sutterella* ↓ *Rhodococcus*, *Phyllobacterium* and *Staphylococcus*	(Cavadas et al., 2020)
Gantuya et al./2020	48 GC, 20 gastritis, 40 atrophy, 40 IM.	GB	16S rRNA gene sequencing	↑ *Lactobacilli*, *Enterococci*, *Carnobacterium*, *Glutamicibacter*, *Paeniglutamicibacter*, *Fusobacterium*, *Parvimonas*	(Gantuya et al., 2020)
Deng et al./2021	25 CG, 34 GC	GB	16S rRNA gene sequencing	↑ Proteobacteria, Firmicutes, Actinobacteria, Bacteroidetes, Fusobacteria	(Deng et al., 2021)
Kadeerhan et al./2021	193 GC	GB	16S rRNA gene sequencing	↑ Actinobacteria, Bacteroidetes, Firmicutes, *Bacillus*, *Capnocytophaga*, *Prevotella*	(Kadeerhan et al., 2021)

CG, Chronic gastritis; GB, Gastric biopsies; GC, Gastric cancer; GJ, Gastric juice; GM; Gastric mucosa; IM, Intestinal metaplasia; NA, Not available.

#### 2.3.4 The intestinal microbiota

The colon has the largest community of bacteria in the human digestive system, with a nearly 10^7^-fold increase in population compared to the stomach (Sender et al., 2016). Crosstalk between *H. pylori* and other commensal bacteria along the gut has a great impact on the gastric and colonic microbiota and may influence the homeostasis of the entire system (Myllyluoma et al., 2007; Serrano et al., 2021). Due to the close interplay between *H. pylori* infection, the gut microbiome, and gastritis, the alterations in the gut microbiota could be associated with chronic gastrointestinal disorders (Ferreira et al., 2018; Yang et al., 2019). *H. pylori* infection leads to the enrichment of Firmicutes, Proteobacteria, Actinobacteria, and Acidobacteria, while reducing the proportion of Bacteroidetes phylum in the intestine of the patients suffering from gastric cancer (Gao et al., 2018a). Long-term infection with *H. pylori* further results in *Akkermansia* overgrowth in the colon (Heimesaat et al., 2014). A study by Wang et al. showed that gastric cancer patients have higher levels of *Lactobacillus*, *Lachnospiraceae*, *Escherichia-Shigella*, *Nitrospirae*, and *Burkholderia* compared to the control group, confirming earlier findings that *Lactobacillus* is widely colonized in gastric cancer (Wang et al., 2016). Although there is still no consensus on the role of the gut microbiome in gastric carcinogenesis, the dysbiotic microbial population may raise the risk for gastric cancer by increasing the inflammatory process in the stomach and inducing immunological responses (Ferreira et al., 2018). Additionally, microbial dysbiosis has a crucial role in tumor initiation and progression by inducing chronic inflammation, dysregulating the immune response, and producing toxins and metabolites (Lertpiriyapong et al., 2014). Several mechanism-oriented studies are required to identify the processes underlying microbial participation in the occurrence of gastric cancer and to prevent carcinogenesis.

## 3 The gut microbiota and the host immune system

It has been well documented that the microbiota in the stomach may have a role in cancer progression and responsiveness to anticancer treatment (Vivarelli et al., 2019). The interplay between the gut microbiota and gastric mucosal immunity is complicated and includes multifold interactions in homeostasis and disease (Zheng et al., 2020). The innate immune system provides the foremost barrier that acts immediately when an antigen enters the body, while the second line of defense against pathogens is the adaptive immune system (Thakur et al., 2019). Toll-like receptors (TLRs) are the main elements of the human innate immune response *and* perform fundamental roles in mediating inflammatory pathways by detecting pathogen-associated molecular patterns (PAMPs) such as LPS, flagellin, and peptidoglycan. Moreover, antigen-presenting cells (APCs), such as dendritic cells (DCs), develop in response to PAMPs via pattern recognition receptors (PRRs). DCs can directly stimulate CD8^+^ T cells and also can provoke naive T cells to generate CD4^+^ T cells, especially CD4^+^ T regulatory cells (Tregs), and Th17 cells. TLR-mediated signaling can trigger the transcription factor nuclear factor kappa-light-chain-enhancer of activated B cells (NF-κB), interferon regulatory factor 3 (IRF3), and mitogen-activated protein (MAP) kinases p38 that initiate downstream signaling pathways (Kawasaki and Kawai, 2014). The main adaptor protein that mediates signals upon stimulation of TLRs is myeloid differentiation primary response gene 88 (MyD88) that enhances the systemic innate immune cell response (Wolz, 2016). MyD88 can recruit IL-1R-associated kinase family (IRAKs) and thereafter activates the MAP3 kinases, which regulates the activation of c-Jun N-terminal kinase (JNK), NF-κB, and MAP kinases p38 (Huang et al., 2004).

It has been elucidated that the gut microbiota oddly modulate the maturation and operation of innate and adaptive immunity. Adaptive immunity is made up of cellular and humoral immunity and is defined by the presence of lymphocytes including T and B cells. The development and function of the adaptive immunity are regulated by gut microbiome, meanwhile, the maturation of the gut microbial community is influenced by the host immune components (Lazar et al., 2018). The impact of the gut microbiota on the maturation of the host immune system were investigated in a study concluding that the microbiota can influence the activity of the gut and systemic immunity, including pathogen removal (Lee and Mazmanian, 2010; Wiertsema et al., 2021). Compared to normal mice models, germ-free (GF) mice, which have no microorganisms living inside or outside them, exhibit defective maturation of gut-associated lymphoid tissues (GALT), in addition to fewer and smaller Peyer’s patches, cellular mesenteric lymph nodes, and less cellular lamina propria of the small intestine. Consequently, the gut microbiota are essential for the development and function of multiple populations of gut immune cells.

The gut microbiota can boost epithelial cytokine expression that controls the activity of T and B lymphocytes, as well as macrophages (Cianci et al., 2019). The host inflammatory response is initiated by expressing particular cytokines such as IL-1β, TNF-α, IL-2, IL-6, IL-15, IL-21, and IL-23, while IL-10 and transforming growth factors-β (TGF-β) secretion can induce the host anti-inflammatory response (Kany et al., 2019). As a result, the inflammatory or homeostatic state of the gut is determined by the equilibrium between the ratios of pro- and anti-inflammatory cytokines.

The host immune system and cancer progression are intricately linked and thereby changes in the balance between the gut microbiota and the host immune response might trigger tumor development (Pagliari et al., 2017; Chen et al., 2021). The development of a single tumor cell into the primary tumor is termed tumor progression. The upregulation of cell proliferation and decreased cell apoptosis, which are enhanced by inflammation-driven mechanisms, have critical impacts on the initial tumor development. Several stimulating impacts of inflammation on carcinogenesis are applied at the level of tumor progression and most of the tumor promoters (Grivennikov et al., 2010).

### 3.1 Specific immune response during *H. pylori* infection and gastric carcinogenesis

*H. pylori* colonization leads to the aggressive stimulation of the innate and adaptive immunity and is a well-understood risk factor for the progression of stomach cancer. Normally, the host’s innate defense is activated when *H. pylori* resides in the stomach epithelium, leading to the secretion of pro-inflammatory and antibacterial agents by the gastric epithelium (Krzysiek‐Maczka et al., 2018). When the host’s prolonged inflammatory response fails to eliminate *H. pylori*, chronic gastritis develops, which may last through the lifespan and cause gastric atrophy, intestinal metaplasia, dysplasia, and intestinal-type gastric carcinoma (Sheh and Fox, 2013). It is suggested that *H. pylori* infection can initiate host inflammation by promoting the secretion of several inflammatory mediators from the gastric epithelium. These cytokines and chemokines attract innate immune cells including neutrophils, macrophages, DCs, natural killer (NK) cells, and lymphocytes (Robinson et al., 2007). IL-8, as a strong chemokine, promotes neutrophils and monocytes infiltration to the gastric mucosal surfaces. Thereafter, the DCs and monocytes induce the release of IL-6, TNF-α, and IL-1β (White et al., 2015). The stimulation of CD4^+^ T cells activity by IL-1β and IL-6 promotes the secretion of different cytokines such as IL-4, IL-5, IL-6, and interferon-γ (IFN-γ). On the other hand, *H. pylori* prevents the proliferation of CD4^+^ T cells by VacA (Beigier-Bompadre et al., 2011).

The reduction in the number of parietal cells developes a state of hypochlorhydria (pH >4) that can perturb the balance of the gastric microbiome. Multiple non-*H. pylori* species such as *Streptococcus* spp., *Lactobacillus* spp., *Xanthomonas* spp., *Proteus* spp., *Klebsiella* spp., *Pseudomonas* spp., *Neisseria* spp., *E. coli*, and *Campylobacter jejuni* have been detected in the stomach of hypochlorhydria subjects (Pereira et al., 2018). The precise processes through which the modified gut microbiome combine with *H. pylori* to cause gastric cancer are yet to be fully elucidated. Multiple studies have reported that the combination of *H. pylori* and other gut microorganisms may work together to raise the risk of stomach cancer. According to an investigation, there is a relationship between harmful shifts in the fecal microbial composition and the elevation in the production of pro-inflammatory cytokines (Wu et al., 2020). The symbiont *Bacteroides fragilis* producing polysaccharide A can downregulate pro-inflammatory cytokines production induced by *H. pylori* colonization (Troy and Kasper, 2010). The colonization of mice with enterotoxigenic *B. fragilis* (ETBF) resulted in the attraction of Th17-dependent myeloid cells to the tumor microenvironment (TME), which aided colon carcinogenesis (Orberg et al., 2017). Commensals in the gut, notably segmented filamentous bacteria (SFB), have been linked to gut immune development and the generation of IL-17 (Hedblom et al., 2018).

*H. pylori* infection has been suggested as the main cause of developing gastric adenocarcinoma so far. The Correa model of *H. pylori*-induced gastric pathogenesis and aging-related alterations in acid production supports the link between disease and age. The cumulative lifetime exposure to reactive oxygen and nitrogen species (RONS), pro-inflammatory cytokines, and tissue damages induced by *H. pylori* are coupled in elderly with a reducing ability to cope with antigens (immunosenescence) and decreased ability to regulate inflammatory responses (inflammaging) (Weiskopf et al., 2009). Immunosenescence, or the age-related reduction in immunity, is manifested in the elderly by diminished antigen presentation, diminished cytotoxic activity, the buildup of effector T cells, reduced naive T cell output and decreased B cell generation. Alterations in the structure of Firmicutes and an expansion in the proportion of Bacteroidetes, have been demonstrated to influence the gut microbiota of the elderly (Vemuri et al., 2018).

### 3.2 Effects of the gut microbiota on gastric cancer immunotherapy

Innate and adaptive immune cells, as well as pro-inflammatory mediators are involved in the initiation and expansion of inflammation (Luster et al., 2005). Moreover, chemokines (CCL2, CXCL12) are critical for recruiting the inflammatory cells to the site of infection. The gut microbiota have been demonstrated to influence the differentiation and function of M2-tumor-associated macrophages (M2-TAMs), tumor-associated neutrophils (TANs), DCs, and CD8^+^ T cells by their products, such as LPS, polysaccharide-dextran, deoxycholic acid (DCA), and SCFAs (Zhao et al., 2021).

The production of SCFAs by the gut microbiota such as butyrate, acetate, and propionate are demonstrated to have a major role in preventing carcinogenesis by stimulating apoptosis and growth arrest in malignant cells (Tang et al., 2011). SCFAs operate as extracellular and intracellular signal mediators and have a major part in the development and function of the host cell, particularly immune cells, attributing to epigenetic alteration and receptor-mediated signaling (Parada Venegas et al., 2019). These metabolites can block histone deacetylases (HDACs) in colonocytes and immune system activity to allow histone hyperacetylation. Similar to the use of HDAC inhibitors, the interaction of mononuclear cells and macrophages with SCFAs leads to reduced production of pro-inflammatory cytokines such as IL-6 and NF-κB. Additionally, by inhibiting HDAC, propionate and butyrate also prevent bone marrow stem cells from transforming into DCs, decreasing excessive inflammatory reactions. By inhibiting HDAC, SCFAs can impact the homeostasis of peripheral T cells and thereby promote the development of Tregs that critically control the intestinal inflammation and carcinogenesis (Furusawa et al., 2013; Arpaia et al., 2013). SCFAs are also involved in the upregulation of immunosuppressive IL-10 and TGF-β cytokines, as well as downregulation of pro-inflammatory cytokines in macrophages and neutrophils, which may lead to inhibition of Th17 cell development, suppression of inflammation, and carcinogenesis. Particularly, butyrate promotes the anticancer effectiveness of oxaliplatin and anti-PD-L1 therapy by directly increasing the cytotoxic CD8^+^ T cell response via ID2-dependent IL-12 signaling through its HDAC inhibitory action. SCFAs can operate either directly on cancer cells as HDAC inhibitors or indirectly by influencing the immune system through certain G-protein-coupled receptors (GPCRs) such as GPR41, GPR43, and GPR109A expressed in colonocytes and immune cells (Koh et al., 2016). Some studies revealed that acetate and propionate might stimulate Tregs in a GPR43-dependent manner, which results in reducing the severity of acute and chronic colitis (Furusawa et al., 2013). Other anti-tumor properties of SCFAs can be associated to their capability to promote mucin secretion and improve the integrity of gut epithelium by initiating the inflammasome and the peroxisome proliferator-activated receptor-γ (PPAR-γ) signaling pathway (Chen and Chen, 2021).

*Bacteroides thetaiotaomicron*, an acetate-producing bacterium, enhances mucus secretion by promoting goblet cell development and mucus-related gene expression (Wrzosek et al., 2013). Moreover, butyrate-producing bacteria might restore vitamin D receptor (VDR) expression in the intestine and reduce the expansion of gut dysbiosis (Nabavi-Rad et al., 2022). Intestinal dysbiosis can enhance the release of LPS, which results in the production of cathepsin K (CTSK) in colorectal cancer (CRC) cells. Recent studies revealed a connection between the overexpression of CTSK and malignancies in colon cancer (Li et al., 2019; Calio et al., 2021). Additionally, CTSK activates the mammalian target of rapamycin (mTOR) pathway by interacting with TLR4 on the macrophage, causing M2 polarization and the generation of cytokines including IL-4 and IL-10. The conversion of M2 to M1 macrophage, a prospective target in cancer immunotherapy, is likely to be aided by the indigenous gut microbiota such as *B. fragilis* (Deng et al., 2016). *B. fragilis* has been reported to enhance the phagocytic functions of macrophages of the innate immune system and polarize them to an M1 phenotype (Frankenberg et al., 2008; Deng et al., 2016). Additionally, *Bifidobacterium bifidum* cell surface polysaccharides can activate the TLR2/MyD88 pathway and also suppress experimental colitis through Treg induction (Verma et al., 2018). As a result, it is plausible that PAMPs generated from the gut microbiota affect PRR, which controls the immune response to gut tumors. Consequently, the activation of NF-κB increases the expression of pro-inflammatory cytokines including TNF-α and IL-6, which might explain why immunosuppressive myeloid cells accumulated in TME (Zhang et al., 2021).

NK cells are effector lymphocytes of the innate immunity that modulate numerous forms of tumors by preventing their progression and development (Levy et al., 2011). NK cells can regulate tumor metastasis owing to their capacity to exert direct cellular cytotoxicity without prior sensitization and to release immunostimulatory cytokines such as IFN-γ. Nevertheless, NK cells show an impaired capability to infiltrate tumors in individuals suffering from cancer. Furthermore, in NK cell-based cancer immunotherapy, correcting NK cell dysfunction is a key requirement for immunotherapies against tumors (Borrego et al., 2016). Multiple medicines including immune checkpoint inhibitors (ICIs) and monoclonal antibodies have been developed to reverse the impaired NK cell function (Wang et al., 2021). A favorable gut microbiome can induce the accumulation of NK cells, which target tumor cells and enhance tumor surveillance. The development of precision medicine requires deep knowledge of the interaction between the gut microbiome and the immune response in cancer immunotherapy (Furusawa et al., 2013). Consequently, intestinal microbiota are attractive targets to manage inflammation-related cancers, as fecal microbiota transplantation (FMT) is becoming a popular way to enhance the gut microbiota and metabolome (Zhao et al., 2021).

**Figure 2:**
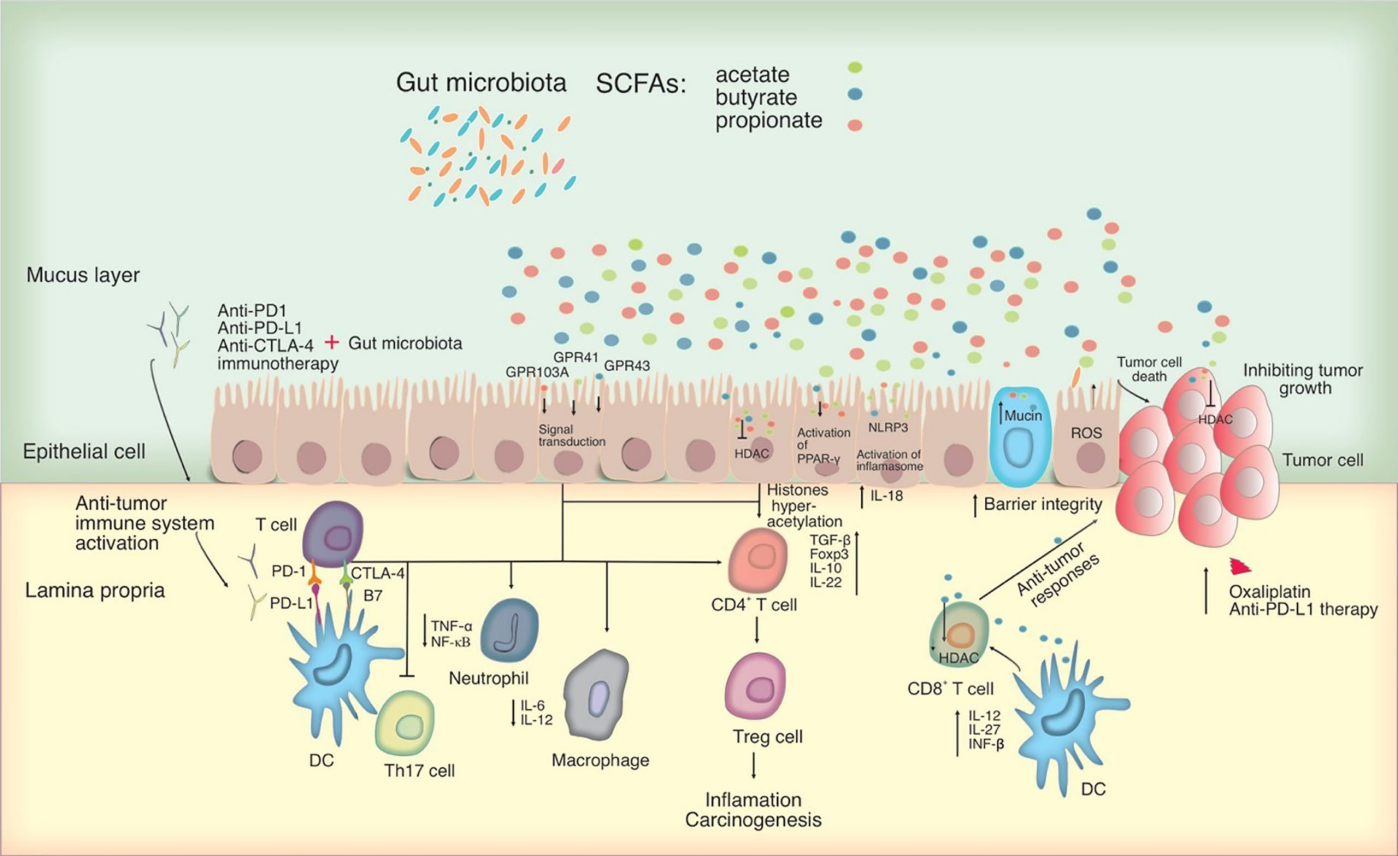
Potential mechanisms for microbiota-mediated immunomodulation in tumor cells. SCFAs, which primarily consist of acetate, butyrate, and propionate can act as an HDAC inhibitor and influencing directly on cancer cells. By interacting with particular GPCRs including GPR41, GPR43 and GPR109A, SCFAs can have an effect on the immune system, leading to upregulation of immunosuppressive IL-10 and transforming growth factor-beta (TGF-β), downregulation of pro-inflammatory cytokines in macrophages and neutrophils, and inhibition of differentiation towards T helper type 17 (Th17) cells, thereby suppressing inflammation and carcinogenesis. SCFAs activate the inflammasome and the PPAR-γ pathway, promoting mucin production and improving epithelial integrity. SCFAs were also shown to activate the NLR family pyrin domain containing 3 (NLRP3) inflammasome, modulating the production of IL-18, which protects epithelial integrity. Significantly, SCFAs, in particular butyrate, may alter CD8^+^ T cell antitumor responses by influencing DC signaling pathways involving IL-12, IL-27, and IFN- β, all of which have an impact on tumor combination therapy. In this figure, the role of PD-1 and CTLA-4 in the priming and effector phases of anti-tumor immune responses is shown. Anti-CTLA-4 blocking antibodies may thereby restore T cell priming in lymph nodes, whereas PD-1 signaling inhibition may allow T cells to operate as tumor effectors. Other cell types in the tumor microenvironment, such as DCs, may also express PD-1 and hence be impacted by PD-1 inhibition. Blocking PD-1 and CTLA-4 may influence T helper cell profiles directly or indirectly by changing the microbiota. HDAC, Histone deacetylase; PD-L, Programmed death-ligand; CTLA-4, cytotoxic T-lymphocyte-associated protein 4; PPAR-γ, peroxisome proliferator-activated receptor-γ; GPCRs, G-protein-coupled receptors.

The identification of proteins that suppress T cell responses such as Cytotoxic T-lymphocyte-associated protein 4 (CTLA-4), programmed death-ligand 1 (PD-1) and its ligand PD-L1 opens up new opportunities for treating certain cancers having elevated production of such proteins. CTLA-4 and PD-1 are commonly found on activated T cells, while PD-1 ligands (PD-L1 and PD-L2) are found on a variety of cell types including antigen-presenting cells, innate immune cells, epithelial cells, and endothelial cells (Seidel et al., 2018). Activation of CTLA-4 and PD-1 can influence the proliferation of tumor-specific T cells and thereby protecting tumor cells in the TME. CTLA-4 controls the immune response just after T cells activation and PD-1 proceeds later to promote T cell death and subsequently terminate the immune response (**Figure 2**). Anti-CTLA-4, PD-1, and PD-L1 inhibitors are the main types of checkpoint inhibitor immunotherapy drugs that have been developed and reported to be an efficient treatment for various malignant tumor types. The anti-PD-L1 inhibitor has been suggested for patients with advanced gastric cancer and positive PD-L1 expression. Ipilimumab is the single Food and Drug Administration (FDA)-approved ICI and the most prominent member of CTLA-4 inhibitor for cancer treatment (Naimi et al., 2022). Furthermore, PD-1 inhibitors nivolumab, pembrolizumab, and cemiplimab, as well as PD-L1 inhibitors atezolizumab, avelumab, and durvalumab are on the current list of authorized medicines (Vaddepally et al., 2020; Twomey and Zhang, 2021). Combining therapy of CTLA-4 and PD-1 blockers with checkpoint inhibitors can boost the immune response by stimulating the signaling pathways that lead to anticancer impact of T cells (Rotte, 2019).

According to new findings, the gut microbiota appears to have a substantial part in regulating responses to immunotherapies (Shui et al., 2020). The link between the gut microbiota and immunotherapy effectiveness was demonstrated by mice experiments, in which particular bacterial taxa were dominated in individuals, before checkpoint blockade therapy (Jain et al., 2021). According to an *in vivo* study, mice colonized with stool isolates from responders had a better outcome than mice colonized with stool specimens from non-responders with checkpoint blockade treatment. Intestinal bacteria appear to be necessary for the best response to cancer chemotherapy and immunotherapy via modulating myeloid cell activity (Hayase and Jenq, 2021). Two elements of the gut microbiota contribution to therapy effectiveness have been suggested, which include an adjuvant impact of translocated bacteria after the therapy-induced disruption of intestinal epithelial barrier and a priming impact given by the gut-inherent microbial community prior to the therapy, which explore the emerging role of microbiota in cancer immunotherapy (Gorjifard and Goldszmid, 2016).

In melanoma mice treated with anti-PD-1/PD-L1 drugs, the genus *Bifidobacterium* was discovered to be the main inducer for enhanced tumor-specific T cell responses and intratumoral CD8^+^ T cells (Shui et al., 2020; Liu et al., 2021). In a clinical trial, *Akkermansia muciniphila* supplementation was demonstrated to promote the efficacy of PD-1 blockade through stimulating the infiltration of CCR9^+^ CXCR3^+^ CD4^+^ T lymphocytes into TME in an IL-12-dependent manner (Routy et al., 2018). The higher proportion of *Bifidobacterium longum*, *Collinsella aerofaciens*, and *Enterococcus faecium* among the gut microbiota was presented with an increased response to PD-1 inhibitors (Matson et al., 2018). Furthermore, longer progression-free survival (PFS) in ipilimumab treatment of melanoma was positively associated with the proportion of *Faecalibacterium* and other Firmicutes bacteria, while negatively associated with the presence of *Bacteroidetes* genus (Chaput et al., 2017). *Bacteroides caccae* has been demonstrated as the main bacteria that present ICIs responders in various types of treatment. Regarding responders treated with anti-CTLA4 and anti-PD-1 immune checkpoint blockade, *Faecalibacterium Prausnitzii*, *Bacteroides thetaiotamicron*, and *Holdemania filiformis* were exhibited as the predominant bacteria (Frankel et al., 2017).

In an animal model of adoptive T cell transfer treatment, the gut microbiota was found to have an adjuvant impact during cancer immunotherapy (Vetizou et al., 2015). The translocation of bacteria into the mesenteric lymph nodes resulted in higher levels of circulating bacterial products, which activated DCs and enhanced the activity of transplanted T lymphocytes (Lathrop et al., 2011). Firmicutes phylum and Ruminococcaceae family are exhibited to be linked to both responses and toxicity, whereas the Bacteroidales order of the Bacteroidetes phylum was linked to a loss of response to immune checkpoint blockade. Taken together, despite the presence of major challenges in addressing the inherent contribution of microbiota in the regulation of cancer immunity, a great deal of evidence held the gut microbiota responsible for modifying the host antitumor immune response and immune checkpoint blockade response (Gopalakrishnan et al., 2018).

## 4 Microbiome-based therapeutic approaches in gastric cancer

It has become apparent that one of the major factors influencing the effectiveness of cancer treatments is the composition of the commensal microbiota (Soto Chervin and Gajewski, 2020). There is growing evidence to show that the interaction of the immune system and the gut microbiota within the host affects how the body reacts to cancer immunotherapy (Ma et al., 2019). Designing therapeutic treatments to improve the patient’s microbial structure is highly recommended, which necessitates an understanding of the mechanisms underpinning the efficacy of microbiome-based therapeutic interventions. Probiotic and/or prebiotic supplementation as well as FMT are considered the pioneer microbiome-modulating approaches, which are further discussed below.

### 4.1 Effects of probiotics on *H. pylori*, the gut microbiota, and gastric cancer

Probiotics have been proven to beneficially influence the gut microbiota, by temporarily or permanently shaping the composition of the gut microbiota (Hemarajata and Versalovic, 2013). Moreover, gut bacteria can interact directly with the host immune cells, triggering both innate and adaptive immune responses by their immune-modulating properties. Consequently, interactions between the gut microbiota and immune cells lead to the maturation of the host immune system throughout the lifespan (Pickard et al., 2017; Zhao and Elson, 2018; Chen et al., 2021). Additionally, probiotics can modify the effect of the gut microbiota on local immunological and inflammatory responses, through the release of particular cytokines such as IFNs, ILs, TNFs, and TGFs (Sang et al., 2010; Ebrahimpour-Koujan et al., 2020). However, probiotics can also interact with DCs, and bridge the innate and adaptive immune system. Intestinal epithelial cells and DCs may respond to the gut microbiota by their PRRs (Raheem et al., 2021). Following probiotic supplementation, the activated DCs induce a favorable immune response, inhibiting Th1-, Th2-, and Th17-mediated inflammatory response. By reducing the production of TLRs by secreting TNF-α inhibitory metabolites and preventing of NF-κB signaling in enterocytes, probiotics can further reduce intestinal inflammation (Raheem et al., 2021). Moreover, lactic acid produced by probiotics has been demonstrated in animal studies to modulate inflammation by regulating pro-inflammatory cytokines (Murosaki et al., 2000). In recent years, adjuvant therapy with probiotics has received significant attention to boost the success of *H. pylori* eradication (Goderska et al., 2018; Keikha and Karbalaei, 2021).

The progression of gastric malignancy is strongly correlated with the expression of CagA oncoprotein, as CagA-positive *H. pylori* This interaction is assumed to be mainly influenced by CagA-mediated overexpression of IL-8 in the gastric mucosa. Some probiotic strains such as *Lactobacillus acidophilus*, *Lactobacillus bulgaricus*, and *Lactobacillus salivarius* have been proved to reduce *H. pylori*-induced IL-8 upregulation (Kabir et al., 1997; Zhou et al., 2008; Yang et al., 2012). Based on recent studies, the administration of probiotics as a co-supplementary material with standard antibiotic regiments substantially increases *H. pylori* eradication rate (Eslami et al., 2019).

*L. salivarius*, *Lactobacillus gasseri*, *Lactobacillus casei* Shirota, *Lactobacillus johnsonii La1*, *Lactobacillus rhamnosus GG*, and *Saccharomyces boulardii* were recognized to have either anti-*H. pylori* properties or anti-inflammatory activity (Hsieh et al., 2012). Recent investigations suggested that probiotics may improve the host immune system and protect against cancer progression (Lu et al., 2021). Furthermore, probiotic co-supplementation with conventional antibiotic regimens can substantially increase the rate of *H. pylori* eradication (Penumetcha et al., 2021). Some probiotic strains can inhibit *vacA* and *flaA* virulence genes in *H. pylori* by producing substances such as reuterin, which is a potent antimicrobial agent expressed by *Lactobacillus reuteri* (Urrutia-Baca et al., 2018; Song et al., 2019). The metabolites produced by the probiotics including PG, surface proteins, secreted proteins, bacteriocins, cell wall polysaccharides, organic acids, hydrogen peroxide, indole, and extracellular vesicles have several favorable effects on the host by triggering various signaling pathways such as mucus secretion in goblet cells (Teame et al., 2020; Sankarapandian et al., 2022). Furthermore, many probiotic strains are expected to enrich the human gut, and particular species, such as *Lactobacillus* spp., can replicate in the human gastric environment and have the ability to prevent *H. pylori* colonization (Ji and Yang, 2020). Probiotics antagonize *H. pylori* through various mechanisms, including (a) secreting antibacterial compounds like organic acids, bacteriocins, and biosurfactants (b) inhibiting the colonization of *H. pylori* by occupying receptor sites or co-aggregation mechanism, (c) supportive role in the intestinal tissues by stimulating mucin synthesis, and (d) modulating the host immune response (Ji and Yang, 2020). Furthermore, these microbiota can express extensive amounts of lactic acid in the gastric lumen following successful colonization. Probiotics also reduce *H. pylori* growth by competing for binding sites or affecting the adhesion mechanism (Urrutia-Baca et al., 2018).

As previously discussed, probiotics have attracted a significant attention in medicine owing to their beneficial effects on different types of gastrointestinal cancers. However, a vast majority of clinical studies lack an appropriate long-term follow-up regarding the effect of probiotic supplementation as a biotherapeutic approach against cancer progression. Therefore, well-designed, randomized, double-blind, placebo-controlled clinical trial investigations are required to elucidate potential probiotic strains and their effective dosages, as an alternative therapy for cancer control (Javanmard et al., 2018). In addition, there are conflicting clinical outcomes concerning the effect of various probiotic strains on *H. pylori* eradication rate. Several studies have indicated no statistically significant difference between the probiotic supplemented group and the control group when it comes to the eradication rate of *H. pylori* (Armuzzi et al., 2001; Cremonini et al., 2002; Nista et al., 2004; Myllyluoma et al., 2005; Cindoruk et al., 2007; Manfredi et al., 2012; Navarro-Rodriguez et al., 2013; Shavakhi et al., 2013). Likewise, a recent meta-analysis study confirmed that the administration of probiotics to standard therapy regiments does not improve the eradication rates of *H. pylori* (Lu et al., 2016a). However, another meta-analysis study reported that probiotic supplements can improve *H. pylori* eradication rate, reduce antibiotic-related side effects, and decrease individual incidences of multiple symptoms (Tong et al., 2007; Zou et al., 2009; Wang et al., 2013; Lu et al., 2016b). It has been further suggested that probiotics may not directly eliminate *H. pylori*, but rather maintain low levels of the pathogen in the stomach; thereby, in combination with antibiotics, probiotics may increase the eradication rate and decrease the adverse effect of antibiotics (Gotteland et al., 2006). Consequently, the main advantage of probiotic supplementation is currently considered the reduction of antibiotic-related adverse effects (Felley et al., 2001; Cremonini et al., 2002; Tursi et al., 2004).

It may be possible to revisit some fundamental concepts about probiotics and concentrate on biologically relevant issues to ease the transition from empirical to patient-oriented therapeutics through high-throughput sequencing and experimental methods. Finally, the development of evidence-based policy should be constructed on large-scale randomized and blinded clinical trials, preferentially without commercial interests (Suez et al., 2019).

### 4.2 Effects of prebiotics on *H. pylori*, the gut microbiota, and gastric cancer

Prebiotics are indigestible food components that beneficially influence the host’s health by promoting the colonization and/or function of one or a small proportion of the gut commensals (Gibson and Roberfroid, 1995; Ziemer and Gibson, 1998; Serban, 2014). Inulin, oligofructose, lactulose, resistant starch, and wheat bran have all been employed in CRC research as prebiotic products. Combinations of probiotics and prebiotics, termed synbiotics, may result in additive or synergistic effects on gut function (Serban, 2014). Isomalto-oligosaccharides (IMO), fructo-oligosaccharides (FOS), galacto-oligosaccharides (GOS), lactulose, and resistant starch (the fraction of starch that resists digestion in the small intestine) are all commercially available prebiotics (Tymczyszyn et al., 2014).

Numerous investigations have looked into the health advantages of fiber ingestion, both with or without the prebiotic effect (Slavin, 2013). Epidemiologic investigations propose that sufficient fiber intake reliably reduces the risk of cardiovascular disease CVD and coronary heart disease (CHD), primarily within a decrease in low-density lipoprotein (LDL) levels (Threapleton et al., 2013). One of the prebiotic mechanisms of action is the growth-promoting impact on members of the *Bifidobacterium* and *Lactobacillus* genera, resulting in greater butyrate, acetate and lactate production (Lordan et al., 2020). SCFA production has several health benefits such as improving the gut barrier function, as well as mineral absorption (Ballan et al., 2020). Moreover, prebiotics similar to probiotics can modify the gut microbiota structure and function in particular on immune responses, pathogenic defense mechanisms, bowel function, and stool consistency (Gibson et al., 2017). The capacity of prebiotics to regulate the host immune responses is mainly by modulating the Th2 and Th1 response (Reid et al., 2003).

In a recent study, patients were supplemented with several probiotic strains including *L. rhamnosus*, *L. casei*, *Streptococcus thermophilus*, *Bifidobacterium breve*, *L. acidophilus*, *Bifidobacterium longum*, and *L. bulgaricus*, and co-supplemented with magnesium as well as fructooligosaccharide as prebiotic. According to this investigation, adding synbiotics to the conventional anti-*H. pylori* regime can help improve drug tolerance, while probiotics plus prebiotics could increase the rate of eradication significantly (Shafaghi et al., 2016).

Prebiotics are presented with an osmotic effect in the gut lumen, which can lead to diarrhea, abdominal pain, and gastroesophageal reflux following a large dose consumption. Moreover, prebiotic fermentation in the colon may cause gaseousness and bloating (Marteau and Seksik, 2004). Therefore, to develop recommendations for cancer prevention and therapy by prebiotic administration, well-designed, randomized, double-blind, placebo-controlled human trials utilizing probiotics and/or prebiotics are required (Serban, 2014).

### 4.3 Beneficial effects of FMT on *H. pylori*, the gut microbiota, and gastric cancer

The emergence of FMT application in various ill states is owing to its significant efficacy in treating recurrent *Clostridioides difficile* infection (rCDI) (Monaghan et al., 2021; Azimirad et al., 2022). The engraftment of microbial composition from a healthy donor to an appropriate patient thoroughly modifies the patient’s gut microbiota shifting it toward the donor’s microbial profile (Wang et al., 2019). SCFA-producing bacteria as well as mucin-producing bacteria such as *A. muciniphila* are reported in several studies to be enriched among the patient’s microbial community following a successful FMT (Ser et al., 2021).

Washed preparation of FMT was applied in a recent pilot study to eradicate *H. pylori* infection. The result of this study indicated the potential capacity of FMT in attenuating *H. pylori* infection to some extent (Ye et al., 2020). Considering the substantial influence of FMT on the intestinal microbiota compared to the gastric microbiota, clinical trials have investigated the impact of FMT on lower gastrointestinal disorders including CRC (Chen et al., 2019). Transplanting stool samples of patients suffering from CRC to GF mice was reported to increase the risk of developing intestinal inflammation and dysplasia in the animal models (Wong et al., 2017). However, the possible influence of intestinal microbiota on the progression of gastric cancer should be noted and animal studies and clinical trials should consider FMT as a stopgap treatment for gastric adenocarcinoma and precancerous lesions of gastric cancer.

## 5 Conclusion and future perspective

Recent advances in culture-independent techniques, mainly high-throughput sequencing and NGS technology, have expanded our understanding of the correlation between the gut microbiota and gastric carcinoma. Current studies have provided solid evidence regarding the effect of *H. pylori* infection on the gut microbial composition, microbial dysbiosis, and cancer progression. It is undoubted that substantial differences exist between the gut microbial composition of patients with gastric pathology, atrophic gastritis, IM, and gastric adenocarcinoma. Consequently, dysbiosis is suggested as a dynamic process that triggers and develops *H. pylori*-associated gastric carcinoma. Furthermore, the major role of the host immune system during *H. pylori* colonization, microbiota alteration, and cancer progression has encouraged researchers to develop innovative therapeutic paradigms in cancer immunotherapy. Pioneer investigations have proposed this therapeutic intervention as an applicable approach, which can be promoted by restoring the inherited microbial composition through probiotic supplementation or FMT. However, some challenges and bottlenecks remain to be addressed about the effective biomarkers to assess the immune condition of patients, the root of therapy failure, and the possibilities for predicting treatment outcomes. Exploring these challenges may shed light on novel treatment directions to reduce the risk of developing therapeutic side effects.

## Author contributions

FF, BA, and AY contributed to the literature review and wrote the draft of the manuscript. AY and FF designed the figures for the manuscript. FF illustrated the figures for the manuscript. AY worked on concept and design of the study, interpreted the collected information and supervised the project. AS and MZ provided clinical advice and guidance for improving of the manuscript. AY, AN-R, and NS critically revised the final version of the manuscript. All authors approved the final version of the manuscript and the authorship list.

## Funding

This study was financially supported by a research grant (no. RIGLD 1128, IR.SBMU.RIGLD.REC.1399.046) from the Foodborne and Waterborne Diseases Research Center, Research Institute for Gastroenterology and Liver Diseases, Shahid Beheshti University of Medical Sciences, Tehran, Iran.

## Acknowledgments

The authors would like to thank the members of the Foodborne and Waterborne Diseases Research Center at the Research Institute for Gastroenterology and Liver Diseases, Shahid Beheshti University of Medical Sciences, Tehran, Iran.

## Conflict of interest

The authors declare that the research was conducted in the absence of any commercial or financial relationships that could be construed as a potential conflict of interest.

## Publisher’s Note

All claims expressed in this article are solely those of the authors and do not necessarily represent those of their affiliated organizations, or those of the publisher, the editors and the reviewers. Any product that may be evaluated in this article, or claim that may be made by its manufacturer, is not guaranteed or endorsed by the publisher.
